# Clinical, Imaging and Procedural Risk Factors for Intrauterine Infective Complications After Uterine Fibroid Embolisation: A Retrospective Case Control Study

**DOI:** 10.1007/s00270-020-02622-2

**Published:** 2020-08-26

**Authors:** Josephine Mollier, Neeral R. Patel, Alison Amoah, Mohamad Hamady, Stephen D. Quinn

**Affiliations:** 1 Medicine, Imperial College London, Exhibition Road, London, SW7 2AZ UK; 2 Radiology department, Imperial College Healthcare NHS Trust, London, UK; 3 Obstetrics and Gynaecology Department, St Mary’s Hospital, Imperial College Healthcare NHS Trust, London, UK; 4 Radiology department, St Mary’s Hospital, Imperial College Healthcare NHS Trust, London, UK

**Keywords:** Fibroids, Uterine fibroid embolisation, Infection, Endometritis

## Abstract

**Introduction:**

This was a retrospective case–control study at a single tertiary centre investigating all UFE procedures between January 2013 and December 2018 for symptomatic fibroids. The aim was to determine the clinical, imaging and procedural risk factors which impact upon the risk of post-uterine fibroid embolisation (UFE) intrauterine infection. Cases were patients which developed intrauterine infection post-procedure, and controls were the background UFE population without infection.

**Methods:**

Clinical demographics, presenting symptoms, uterine and fibroid characteristics on imaging and procedural variants were analysed. A *p* value of less than 0.05 was considered statistically significant. The main outcome measures were presence of infection and requirement of emergency hysterectomy.

**Results:**

333 technically successful UFE procedures were performed in 330 patients. Infection occurred after 25 procedures (7.5%). 3 of these patients progressed to overwhelming sepsis and required emergency hysterectomy. Clinical obesity (BMI > 30) (OR 1.53 [1.18–1.99]) and uterine volume > 1000cm3 (2.94 [1.15–7.54]) were found to increase the risk of infection

**Conclusions:**

UFE is generally safe in patients with symptomatic fibroids. Obese patients (BMI > 30) and those with large volume uteri (> 1000cm^3^) are at slight increased risk of developing infection and require appropriate pre-procedural counselling, as well as careful post-UFE follow-up. BMI and uterine volume may be useful to assess before the procedure to help to determine post-UFE infection risk.

**Electronic supplementary material:**

The online version of this article (10.1007/s00270-020-02622-2) contains supplementary material, which is available to authorised users.

## Introduction

Uterine fibroid embolisation (UFE) is a minimally invasive treatment for women with symptomatic fibroids. The procedure has comparable efficacy to surgical myomectomy and hysterectomy in large randomised control trials, with the added benefits of shorter hospital stay as well as the elimination of surgery and anaesthesia-related risks [1–3]. Patient satisfaction rates for UFE are also high, with enhanced mid and long-term quality of life improvement, as well as excellent efficacy with mean uterine and dominant fibroid volume reduction of 42.8% and 48.8%, respectively [4]

Studies have reported infection as an important complication of UFE, the described incidence ranging from 1.2–17% [1, 5] Although most cases are self-limiting, intrauterine infection can be severe requiring aggressive treatment to avoid sepsis and may necessitate emergent hysterectomy in 0.25–1.6% of cases [6].

Although studies have suggested potential risk factors for post-UFE infection, findings are conflicting, and many have used sample sizes too small to reach statistical significance. The aim of this study is to determine the clinical, imaging and procedural risk factors associated with post-UFE infection.

## Methods

### Patient Group

Patients who underwent UFE at our institution between January 2013 and December 2018 were included. All patients were seen in a specialist fibroid clinic by a gynaecologist where they underwent a full clinical history and gynaecological examination. Magnetic resonance imaging (MRI) or ultrasound imaging was used to assess the fibroid burden. Patients were eligible for UFE if they had symptomatic fibroids and were subsequently referred to interventional radiology for further assessment and counselling. Following UFE, patients were admitted to a gynaecology ward for post-procedure observation. Demographic data collected included age at procedure, body mass index (BMI), parity, presence of heavy menstrual bleeding (HMB) and pressure symptoms. Due to the retrospective nature of this study, some patients were omitted from analysis due to incomplete data.

### Imaging Analysis

Pre-UFE MRI scans were analysed. Parameters collected included pre-procedure uterine volume, diameter of dominant fibroid, number of fibroids present, dominant fibroid type, presence of fibroids with submucosal components and length of the fibroid load in the endometrial cavity.

Uterine volume was assessed by using the ellipsoid formula, as established previously by Volkers and colleagues [3]. Maximal dominant fibroid diameter was determined in either sagittal or axial plane. Submucosal fibroid load was evaluated by measuring the length of all submucosal fibroids added together within the endometrial cavity.

Anterior wall fat (AWF) was used a surrogate measurement of BMI in patients who did not have height and weight measurements recorded. AWF measured at two positions on T2-weighted MRI, where AWF_1_ was defined as the thickness of AWF 3 cm below the umbilicus and AWF_2_ was the largest measurement between the umbilicus and the superior most aspect of the pubic bone (Figure S1). AWF was not measured in MRIs which did not include the umbilicus or pubic bone.

### Definitions and Outcome Data

Technical success was defined as occlusion of at least one uterine artery to 10 heart beats stasis. Post-UFE infection is defined by pelvic pain, fever, vaginal discharge and biochemical or histological evidence of infection. Patients with proven urinary tract infection were excluded. Infection was distinguished from post-embolisation syndrome (PES) by high-grade fever and presentation or symptom persistence greater than 10 days post-procedure, as stated in guidelines by the Royal College of Obstetrics and Gynaecologists [7]. PES was therefore defined as fever resolving within 10 days of the procedure. In patients with post-UFE infection, the length of hospital stay, requirement for re-admission, markers of infection (fever, serum white cell count, c-reactive protein, high vaginal swabs and blood cultures), improvement with intravenous antibiotics and any subsequent re-intervention required to treat the infection were recorded.

### Statistical Analysis

SPSS software version 25 (IBM, New York, US) was used for statistical analysis. Spearman’s rank was used to assess the correlation between both anterior wall fat measurements and any BMIs available in the patient notes. A regression line was drawn for estimation of BMI from AWF. Kolmogorov–Smirnov tests were used to assess for normality. Mann–Whitney U tests were used for comparison of medians of continuous variables, and Chi-squared tests of independence were used for comparison of categorical variables as the data were nonparametric. Logistic regression was then used for multivariate analysis of any factors which significantly affected infection risk on univariate analysis. Statistical significance was defined as *p* < 0.05.

## Results

336 procedures were performed in 333 patients. Three patients were excluded due to failure to complete the procedure. The average hospital stay was 2.5 ± 3.81 days. Three patients had repeat UFE (two due to initial incomplete devascularisation and one patient had right and left arteries embolised on separate occasions due to a large fibroid load).

Height and weight measurements were available for 117 patients, for calculation of BMI. Both measurements of AWF_1_ and AWF_2_ had strong correlations with BMI which were statistically significant, *R* = 0.70, *p* < 0.001 and *R* = 0.76, *p* < 0.001, respectively (Table S1). As AWF_2_ had a stronger correlation, this measurement was used to estimate BMI (Figure S2). MRIs were not available for 47 patients, and so 286 patients were included for analysis of imaging factors. Intrauterine infection occurred after 25 procedures (7.5%). 4 infections presented during initial hospital stay and persisted for longer than 10 days, and 21 occurred after discharge.

### Baseline Characteristics

The baseline characteristics are summarised in Table 1. Thirty-nine patients who underwent UFE before 2014 were embolised with tris-acryl gelatin spheres (Embozene, Varian, California, US), but as these are no longer used in UFE at our site, these patients were excluded from analysis of procedural factors. The median AWF and uterine volume were significantly higher in the infection cohort compared to the background UFE population. There were no statistical differences in age, parity, presenting symptoms, number of fibroids, dominant fibroid diameter (defined as the largest fibroid in a particular patient), presence of a submucosal component, fibroid load in the endometrial cavity and amount or size of embolisation particles used in the infection group compared to the background UFE population. Statistical analysis was not performed for ethnicity or dominant fibroid type, due to small numbers in the infection cohort.

**Table 1:** Baseline characteristics in the post-UFE infection group vs the background UFE population

Characteristic	Infection patients (*n* = 25)	Background UFE population	*P* value
Age, *n* = 333	49 (44–53)^+^	48 (45–51)^+^	0.580^a^
Parity, *n* = 301			
Nulliparous	5 (23%)	120 (43%)	0.102^b^
Parity ≥ 1	17 (77%)	159 (57%)	
Presence of menorrhagia, *n* = 326			
Yes	23 (92%)	259 (86%)	0.775^c^
No	2 (8%)	42 (14%)	
Presence of pressure symptoms, *n* = 319			
Yes	18 (78%)	222 (75%)	0.120^c^
No	5 (22%)	74 (25%)	
Anterior wall fat (cm), *n* = 234	4.5 (2.8–6.6)^+^	3.2 (2.5–4.0)^+^	**0.012**^a^
Number of fibroids, *n* = 286			
≤ 8 fibroids	13 (59%)	137 (52%)	0.669^b^
> 8 fibroids	9 (41%)	127 (48%)	
Dominant fibroid diameter (cm), *n* = 286	7.8 (5.8–11.0)	7.0 (5.5–9.5)	0.181^a^
Dominant fibroid type, *n* = 286			
Submucosal	2 (9%)	16 (6%)	N/A
Intramural	14 (64%)	187 (71%)	
Subserosal	6 (27%)	61 (23%)	
Presence of a submucosal component, *n* = 286			
Yes	9 (41%)	115 (44%)	0.986^b^
No	13 (59%)	149 (56%)	
Fibroid load in the endometrial cavity (cm), *n* = 286	0 (0–1.70)^+^	0 (0–2.53)^+^	0.583^a^
Uterine volume (cm^3^), *n* = 286	1165 (540–1721)^+^	825 (527–1204)^+^	**0.049**^a^
Amount of embolisation agent (vials), *n* = 179			
≤ 4 vials	5 (38%)	105 (63%)	0.141^b^
> 4 vials	8 (62%)	61 (37%)	
Size of embolisation particles (microns),*n* = 255			
≤ 500	14 (70%)	182 (77%)	0.630^b^
> 500	6 (30%)	53 (33%)	

^**+**^denotes where continuous data presented as median (interquartile range), all other categorical data are presented as number of patients (percentage). The number of patients for which data were available is indicated in the first column

^a^Denotes where Mann–Whitney-*U* tests were used for statistical analysis

^b^Denotes where Chi-squared tests on independence used for statistical analysis. Yates’ continuity correction was used when there were less than 3 categories. ^**c**^Denotes where Fisher exact test was used

Due to an incomplete data set, not all patients could be included in all analyses

With regard to outcomes, the median hospital stay for the infection cohort was 7 days, (interquartile range 4–14 days), compared to 2 days (interquartile 1–2 days) in the background UFE population. This was statistically significant (*p* < 0.001). The readmission rate for infection patients was also significantly higher than the background UFE population, at 84% compared to 2% (*p* < 0.001) (Table 2).

**Table 2:** Number of infection cases in the healthy/overweight category vs obese category

Body mass index	Infection patients (*n* = 22)	Background UFE population (*n* = 212)	*P* value^b^
Healthy or overweight	10 (45%)	159 (75%)	0.007^a^
Obesity (class 1 & 2)	12 (55%)	53 (25%)	

^a^reflects statistically significant result

^b^Chi-squared test of independence used

### Anterior Wall Fat

AWF data for BMI estimation were available for 234 patients. Patients were classified into healthy, overweight, class 1 obesity and class 2 obesity, as per the World Health Organisation’s BMI classifications [25]. With increasing AWF, there is a trend towards increasing proportion of UFE procedures which result in infection (Figure S3). Statistical analysis was not performed due to small numbers in the healthy BMI group.

The rate of infection was significantly higher in the obese cohort compared to the healthy/overweight cohort (Table 2).

### Uterine Volume

Uterine volume ranged from 97 cm^3^ to 2877 cm^3^. The fourth uterine volume quartile has a significantly increased number of post-UFE infections compared to the first quartile (Figure S4).

There was a significantly increased number of post-UFE infection patients in the > 1000 cm^3^ uterine volume group compared to those with uterine volume less than or equal to 1000 cm^3^ (Table 3).

**Table 3:** Infection cases using large uterus criteria of > 1000 cm^3^

Characteristic	Infection cases (*n* = 22)	Background UFE population (*n* = 264)	*P* value^b^
Uterine volume			
≤ 1000cm^3^	8 (4.5%)	169 (95.5%)	0.019^a^
> 1000cm^3^	14 (12.8%)	95 (87.2%)	

^b^Chi-squared test of independence used

^a^reflects statistically significant result

### Dominant Fibroid Diameter

Dominant fibroid diameter ranged from 1.5 cm to 21 cm. There was no obvious relationship between increasing fibroid diameter and number of infections (Figure S5). Statistical analysis was not performed between quartiles due to small numbers of infection cases in each group. The giant fibroid cohort, defined as dominant fibroid diameter larger than 10 cm, was not significantly different to the infection rate in the ≤ 10 cm fibroid cohort (*p* = 0.128).

### Multivariate Analysis

Both BMI ≥ 30 and uterine volume > 1000cm^3^ were significant predictors of developing a post-UFE infection on multivariate analysis. The odds ratio of developing post-UFE infection was 1.53 (95% CI:1.18–1.99) in the obese BMI group and 2.94 (95% CI: 1.15–7.54) in the > 1000cm^3^ uterine volume group, compared to the background UFE population.

### Emergency Hysterectomy Cases

Emergency hysterectomy was performed after three UFE procedures (0.9%). The median age, anterior wall fat, parity, uterine volume, number of fibroids, dominant fibroid diameter and fibroid load in the endometrial cavity were higher in the hysterectomy group compared to the background UFE population. Due to small numbers, statistical analysis could not be carried out. None of the three patients had known previous history of pelvic infection or inflammatory disease, sexually transmitted disease or immunodeficiency. All three patients’ BMI were classified as obese; the rate of emergency hysterectomy in the obese BMI group was 4.6% and 0% in the healthy/overweight group. Two of the three emergency hysterectomies were carried out in patients with uterine volume > 1000cm^3^. The rate of emergency hysterectomy in the ≤ 1000cm^3^ uterine volume group was 0.6%, compared with a rate of 1.8% in the > 1000cm^3^ uterine volume group.

### Clinical Summaries of Emergency Hysterectomy Patients

Details of patients undergoing emergency hysterectomy post-UFE are summarised in Table 4.

**Table 4:** Characteristics of patients undergoing emergency total abdominal hysterectomy (TAH) following UFE

Patient	BMI	Uterine Vol. (cm^3^)	Fibroid diameter (cm)	Outcome
1	44.06	490	5.8	Readmitted 3 days post-UFE *E. coli* infection initially responding to IV antibiotics TAH performed 21 days post-UFE
2	46.36	2037.2	16.6	Readmitted 6 days post-UFE *E. coli* sepsis diagnosed TAH performed
3	Not recorded	1717.4	10.9	readmitted 2 days post-UFE Spiking temperatures failed to resolve with IV antibiotics TAH performed 20 days post-UFE

## Conclusion

### Main Findings

In our study population, patients with increased BMI or uterine volume > 1000cm^3^ had significantly higher risk of developing an infection post-UFE compared to the control UFE population. Such factors should be taken into consideration when offering UFE to patients, and in counselling women regarding the risks of UFE.

Obese patients were found to have 1.53 times (CI: 1.18–1.99) greater risk of developing infection. The median anterior wall fat measurement for patients requiring emergency hysterectomy after uterine sepsis was also notably greater than the control UFE population. The literature regarding obesity as a predictor for post-UFE infection specifically is sparse, but studies investigating surgical patients have found patients with high BMI are at higher risk of developing infection post-procedure [8]. There is also evidence to suggest that patients with infective complications and increased BMI are more likely to have worse outcomes when compared to patients with healthy BMI (BMI < 25) [9]. This is supported by laboratory observations that inflammatory response, production of inflammatory cytokines and microvascular integrity is dysregulated in obese patients [10]. Microvascular dysfunction has a key role in the pathophysiology of sepsis [11], and therefore obese patients may be more likely to develop septic uteri requiring hysterectomy. AWF may in fact be a better predictor of post-UFE infection risk than BMI, as BMI measured using height and weight can be exaggerated by larger uteri.

Increased fibroid burden may lead to an increased volume of devascularised, necrotic tissue post-embolisation, which may lead to an increased risk of infection [12]. In this study, uterine volume, and not dominant fibroid diameter, was shown to have a significant effect on the risk of post-UFE infection. While high uterine volume can be due to a giant fibroid, using dominant fibroid diameter as an indicator of the fibroid load does not account for patients with innumerate small fibroids. The current study reports three times greater odds of infection, as well as a 1.8% risk of emergency hysterectomy, in patients with larger uteri, compared to patients with uteri less than 1000cm^3^. The presence of a giant fibroid did not significantly increase infection risk. This suggests that UFE is a safe treatment option for women with giant dominant fibroids.

Improved identification of high-risk patients will lead to better preparation from medical personnel involved in the care of these patients regarding infection risk, and therefore earlier diagnosis and initiation of aggressive treatment. Earlier antibiotic treatment may reduce the need for emergency hysterectomy due to sepsis. Additionally, pre-procedure weight loss could be utilised to reduce post-procedure hysterectomy risk. Another possible way to minimise infection risk may be to perform a two-step embolisation, one side at a time, for high risk women. This was done for one lady in our study, but further research is required to determine if this technique effectively mitigates the infection risk.

### Strengths and Limitations

The strengths of this study include the large sample size. The elliptical formula used to estimate uterine volume can be applied to both MRI and ultrasound imaging. As a result, it can be used by centres where MRI is not performed routinely for fibroid uteri. It can also be calculated easily and rapidly at the point of care, allowing for rapid assessment of a patient’s post-procedure infection risk. All 24 infection patients in this study had high-grade fever and either presented or had symptom persistence beyond 10 days post-UFE, ensuring PES cases were not incorrectly characterised as infection.

There are several limitations to this study. A clear limitation is the low number of patients in infection cohort, and therefore the obesity subgroup and the uterine volume > 1000cm^3^ groups. However, this was unavoidable as infection is an important, but relatively uncommon complication of UFE. Distinction between PES and infection in the absence of positive blood or high vaginal culture remains, to a certain degree, speculative. We followed 10-day cut-off as the main distinction factor given the majority of PES cases are expected to improve by this time period. As this is a retrospective study, it is conceivable that some patients may have subsequently developed infection and would not have been captured in the relatively short follow-up period. It is possible that some patients presented with infection at other institutions. However, an overwhelming majority of the treated patients were referred from within our centre’s network region and notification from treating physicians for such an important complication would be expected.

The ellipsoid formula used in this study has been widely used in previous fibroid studies and is quick and easy to calculate. However, fibroids often cause irregularity to the uterine contour, causing deviation from a perfect ellipse. Furthermore, this method has also been shown to be subject to intra-observer variability, which may lead to inaccurate measurements [13].

### Interpretation

Several studies have identified a large fibroid load may increase the risk of post-UFE complications, and both cases of fatal sepsis in the literature occurred in women with large uteri [6, 14]. However, a retrospective study of 121 patients found that uterine volume > 750cm^3^ did not increase the risk of infection [15] and a study of 20 patients with “megauteri” (uterus measuring > 1600cm^3^) reported only one proven infection and no emergency hysterectomies [16]. Some studies have found trends towards an increased infection risk in patients with large dominant fibroids (23, 36, 50), but these studies do not report statistical significance. Other investigators have found no change in infection risk due to large fibroid diameter [1, 17]. A recent meta-analysis concluded that UFE is safe in patients with giant fibroids, but there was an overall significantly increased rate of major complications in this group [18], so care must be taken in diagnosing complications early. There has been controversial evidence regarding the risk of infection in patients with submucosal fibroids [19, 20]. The current study shows no significant correlation between fibroid submucosal location and infection.

A volume of 1000cm^3^ is equivalent to a uterus of 24 weeks gestation, which can be assessed in clinic easily using fundal height measurements. This allows for quick and easy identification of potentially high-risk patients. This increased risk should be weighed against the benefit of performing UFE prior to myomectomy as a means to shrink fibroid volume [21]. It is therefore important to consider the risk of infection post-myomectomy in patients with higher BMI, although this has not been specifically studied. Cinar et al. found that there was an increased risk of post-op fever and wound infection, but not emergency hysterectomy in obese patients [22].

A number of factors may explain the differences between the results of this study and previously reported results from retrospective studies and randomised controlled trials. Firstly, many studies included fewer patients with uterine volumes above 1000cm^3^, and some investigators excluded patients with > 1000cm^3^ from studies [23]. The current study found no significant increase in post-UFE infection in patients embolised with more than 4 vials of embolic agent. Prior studies have found increased risk of febrile complications in this cohort [24]. This could be explained by the lack of clear criteria for distinguishing true post-UFE infection from post-embolisation syndrome.

## Conclusion

In conclusion, the findings of this study suggest that BMI, estimated using anterior wall fat, as well as uterine volume may be useful predictors for infective complications post-UFE. Further studies are required to validate these findings and allow for such risk factors to be assessed and modified in clinical practise.

## Electronic supplementary material

Below is the link to the electronic supplementary material.

Supplementary file1 (DOCX 3829 kb)
