# Supplementary material for: Clinical, Imaging and Procedural Risk Factors for Intrauterine Infective Complications After Uterine Fibroid Embolisation: A Retrospective Case Control Study
Source: Cardiovasc Intervent Radiol. 2020 Aug 26;43(12):1910–7. doi: 10.1007/s00270-020-02622-2 (PMC7649153; doi:10.1007/s00270-020-02622-2)
Supplement: Supplementary file 1 — Supplementary file1 (DOCX 3829 kb) [file 270_2020_2622_MOESM1_ESM.docx]

**Supplementary information**

| **Anterior wall fat** | **R_s_ value** | **Co-efficient of determination (r^2^)** |
| --- | --- | --- |
| AWF_1_: 3cm below the umbilicus | 0.70** | 0.49 |
| AWF_2_: thickest measurement between the pubic bone and L1 | 0.76** | 0.58 |

**Table S1:** R values and co-efficient of determination for correlation between AWF_1_ and AWF_2_ measurements and body mass index. Spearman’s rank test was used as the data is non-parametric. **denotes that correlations were significant at the p<0.01 level.

| **Dominant fibroid diameter** | **Number of infection cases (n=22)** | **Background UFE population (n=264)** | **P-value^a^** |
| --- | --- | --- | --- |
| ≤10 cm  >10 cm | 14 (6.2%)  8 (13.1%) | 211 (93.8%)  53 (86.9%) | 0.128 |

**Table S2:** Number of infection cases in patients with large dominant fibroid diameter (defined as >10cm, as previously defined by Rajan et al.) vs the background UFE population. ^a^Chi-squared test of independence used.


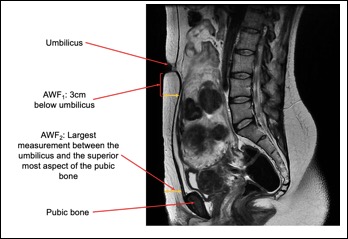


**Figure S1:** Pre-UFE T2 weighted MRI, sagittal view, showing the two measurements of anterior wall fat.


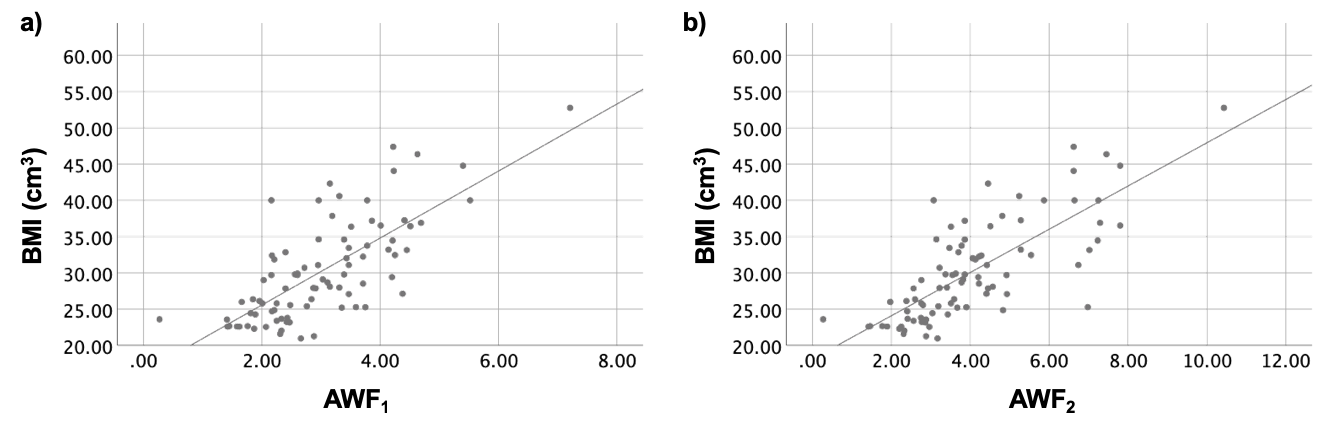


**Figure S2a:** Scatter plot for AWF_1_ (measured at 3cm below the umbilicus) plotted against BMI, including the regression line.

**Figure S2b:** Scatter plot for AWF_2_ (measured at the thickest point between the umbilicus and the superior most aspect of the pubic bone) against BMI, including the regression line.


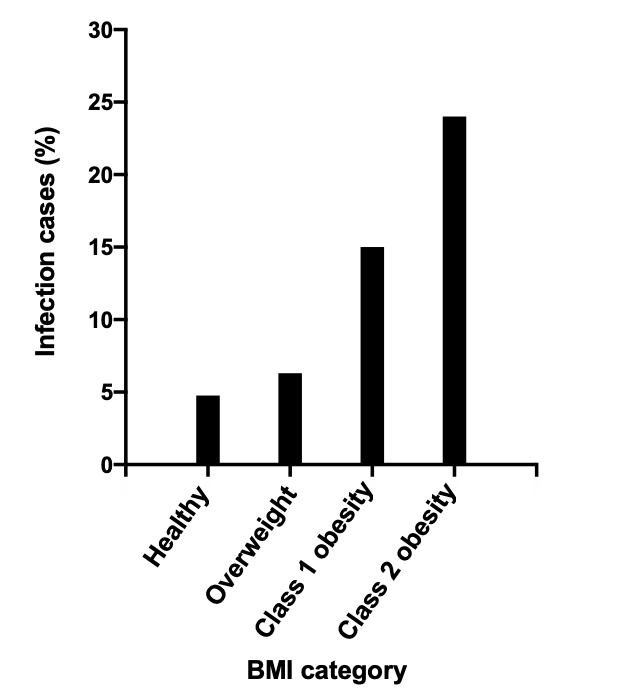


**Figure S3:** Percentage of post-UFE infection cases within each BMI group. BMI estimated using anterior wall fat.


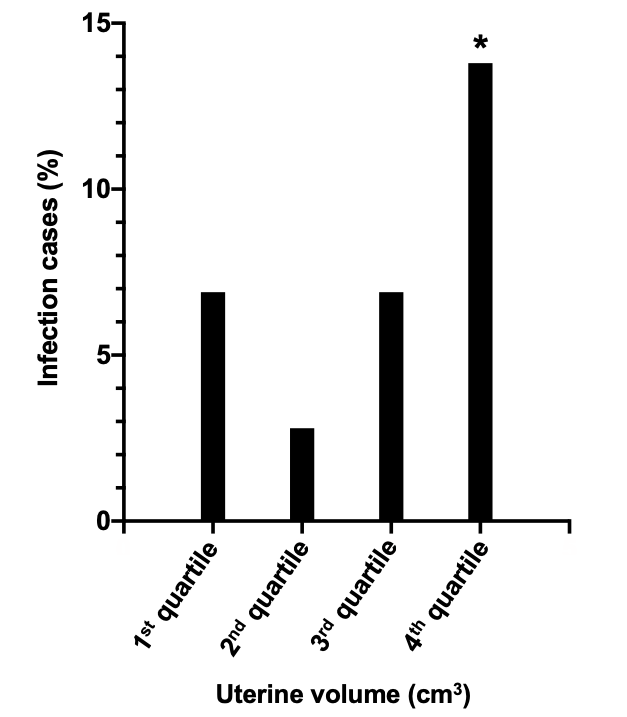


**Figure S4:** Percentage of post-UFE infection cases with increasing uterine volume, Chi-squared test of independence used for statistical analysis and * denotes p<0.05, as compared to the first quartile.


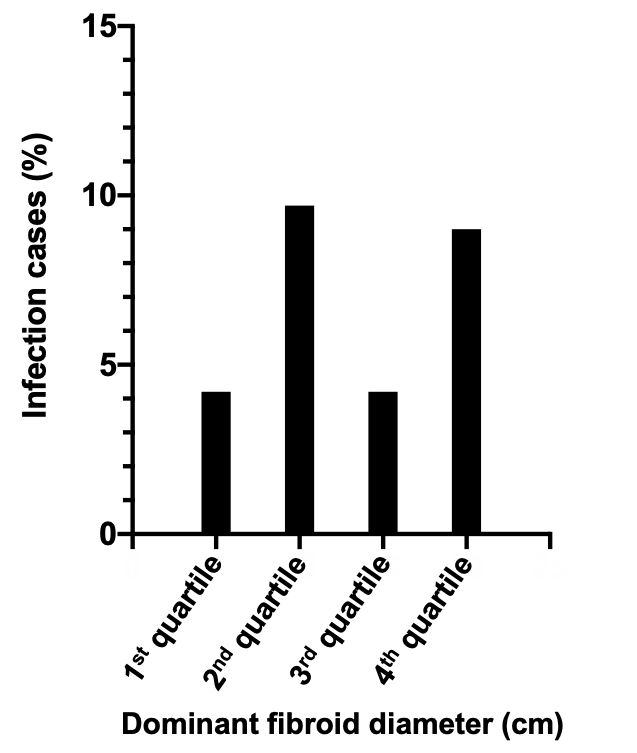


**Figure S5:** Percentage of post-UFE infection cases with increasing dominant fibroid diameter
